# Discovery and quantification of a widespread methane ebullition event in a coastal inlet (Baltic Sea) using a novel sonar strategy

**DOI:** 10.1038/s41598-020-60283-0

**Published:** 2020-03-10

**Authors:** A. Lohrberg, O. Schmale, I. Ostrovsky, H. Niemann, P. Held, J. Schneider von Deimling

**Affiliations:** 1 Christian-Albrechts-Universität zu Kiel, Institute for Geosciences, Marine Geophysics & Hydroacoustics, Otto-Hahn-Platz 1, 24118 Kiel, Germany; 2 Leibniz Institute for Baltic Sea Research Warnemünde, Trace Gas Biogeochemistry, Seestraße 15, 18119 Rostock, Germany; 3 Israel Oceanographic and Limnological Research, Yigal Alon Kinneret Limnological Laboratory, Migdal, Israel; 4 NIOZ Royal Netherlands Institute for Sea Research, Department of Marine Microbiology and Biogeochemistry, Den Burg, The Netherlands, Texel, The Netherlands; 5 Department of Earth Sciences, Faculty of Geosciences, Utrecht University, Utrecht, The Netherlands

**Keywords:** Climate-change impacts, Ocean sciences

## Abstract

How much of the greenhouse gas methane is transported from the seafloor to the atmosphere is unclear. Here, we present data describing an extensive ebullition event that occurred in Eckernförde Bay, a shallow gas-hosting coastal inlet in the Baltic Sea, in the fall of 2014. A weak storm induced hydrostatic pressure fluctuations that in turn stimulated gas ebullition from the seabed. In a finely tuned sonar survey of the bay, we obtained a hydroacoustic dataset with exceptionally high sensitivity for bubble detection. This allowed us to identify 2849 bubble seeps rising within 28 h from the seafloor across the 90 km² study site. Based on our calculations, the estimated bubble-driven episodic methane flux from the seafloor across the bay is 1,900 μMol m^−2^ d^−1^. Our study demonstrates that storm-associated fluctuations of hydrostatic pressure induce bulk gas-driven ebullitions. Given the extensive occurrence of shallow gas-hosting sediments in coastal seas, similar ebullition events probably take place in many parts of the Western Baltic Sea. However, these are likely to be missed during field investigations, due to the lack of high-quality data acquisition during storms, such that atmospheric inputs of marine-derived methane will be highly underestimated.

## Introduction

Methane is an important greenhouse gas, ranking second in radiative forcing by well-mixed greenhouse gases^1^ and with an estimated global net atmospheric emission of about 592 Tg per year^2^. A sudden increase of atmospheric methane from possibly biogenic sources in the past decade has been reported, that has the potential to challenge the intergovernmental goals for the reduction of greenhouse gas emissions as set out in the UN Paris Agreement^3^.

Even though the marine environment hosts massive amounts of methane in the sediment^4^, this system represents a very modest source of atmospheric methane (6–12 Tg CH4 per year)^5,6^. Methane can be transferred from the seabed into the water column by porewater-seawater diffusion, fluid flow, or the ebullition of gas bubbles.

At the seabed-water interface, anaerobic and aerobic microbial oxidation of methane efficiently reduces the dissolved methane fluxes from the sediment into the overlying water column^7,8^. Methane gas bubbles can bypass this microbial sink but their rapid dissolution as they rise in the water column^9^ and the subsequent microbial turnover of dissolved methane result in a highly reduced methane flux to the atmosphere^10^. However, in shallow depth the efficiency of the water column filter is diminished due to the limited retention time of bubbles in the water column and short diapycnal barriers. Therefore, shallow coastal regions can be considered a significant source of atmospheric methane^9,11,12^.

The high sedimentation rates of organic matter that typically occur in coastal regions drive methanogenesis in the seabed. As a result, methane inputs into the atmosphere from these regions are much more substantial than those from open waters^5,13–15^. Moreover, among these coastal sites, the shallow gas-bearing sediments extensively found in river deltas, embayed coastal areas, and estuaries^16–27^ are expected to expand due to global warming^28,29^. Nonetheless, while gas seepages from local geological hot spots of methane emission have been frequently reported^30–40^, widespread methane ebullition from coastal shallow-gas-bearing regions has been poorly investigated.

An accurate estimate of the strength of the atmospheric methane source originating from seabed seepage requires a precise quantification of the methane gas bubble flux through the water column as well as measurements of the methane concentration at the sea surface, but in many cases both are unavailable in sufficient temporal and regional resolution^41^. The problem is largely one of feasibility, since most field campaigns are subject to temporal and spatial restrictions and thus focus on geological hot spots. This strategy necessarily leads to a mismatch of top-down and bottom-up estimations when upscaled for larger areas^42^.

**Figure 1:**
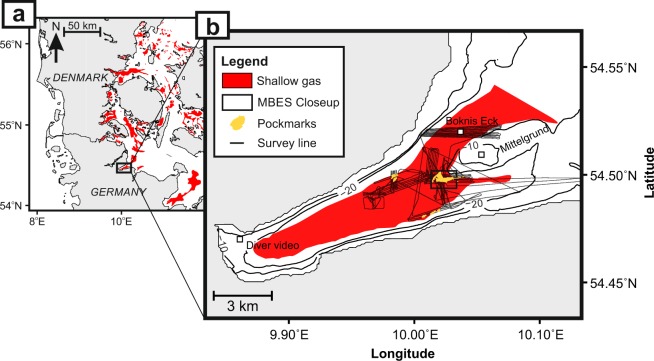
Distribution of shallow gas in the Western Baltic Sea and in the survey area (after Laier & Jensen^17^ and Whiticar^18^). (**a**) Shallow gas identified by acoustic turbidity in the Western Baltic Sea;^17^ (**b**) The distribution of shallow gas in the study area (sediments characterized by zones with acoustic turbidity); figure based on results of Whiticar^18^. The grey lines indicate the survey lines during expedition AL447. The diver video location and the sampling sites of the long time-series of Boknis Eck are also shown. The black box indicates the region of the multibeam echosounding (MBES) closeup (Fig. S1).

Shallow gas has been detected in many areas of the Western Baltic Sea (Fig. 1a). Our study concentrated on the coastal inlet of Eckernförde Bay, one of the world’s best-studied marine sites hosting shallow gas (Fig. 1b). Eckernförde Bay is a typical post-glacial sedimentary basin representative of those often found in high-latitude coastal systems^43^. The pockmarks in the centre of the bay (Fig. S1) were formed by focused groundwater flow^44,45^. Gas ebullition from the pockmark area has been reported in sonar studies^46,47^. However, widespread natural gas ebullition outside of the pockmark area, from gas-bearing sediments across the bay, remains to be proven. Methane entering the water column from the Eckernförde Bay seabed via diffusive transport is effectively filtered by anaerobic oxidation^48–50^. In the present study we employed a novel survey strategy to demonstrate that this microbial filter can be temporarily bypassed during storms, resulting in extensive methane ebullition events of relatively large magnitude.

We hypothesized that gas ebullition in these shallow-gas-bearing regions is controlled by pressure changes on the seafloor and that they are widespread in the bay (Fig. 1b: shallow gas), irrespective of the pockmarks as a geological hot spot (Fig. 1b: pockmarks). Pressure changes on the seafloor are triggered by tidal-induced sea level shifts or wind events. For instance, a correlation between pressure and gas ebullition at marine^33,50,51^ and limnic^52–54^ methane ebullition sites has been described and is supported by theoretical considerations^55^. To test our hypotheses in Eckernförde Bay, we sailed across wide parts of the bay during a storm period in October 2014 that resulted in significant changes in water level (±0.5 m) and air pressure (±1500 Pa). As the working area was sheltered from waves and wind by the bay, a novel survey strategy using dedicated near-stationary sonar imaging was possible. In addition, our survey of the 2 × 4 nautical mile area was conducted at a very low survey speed (approx. 1 kn), and covered 93 nautical miles overall (Fig. 1b). The survey lines were defined based on ship-times equally distributed across both pockmarks and morphologically featureless Holocene mud hosting shallow gas accumulations. Using this exceptionally low survey speed and the fine-tuned sonar settings (see Methods for details), we were able to acoustically probe the water column for gas bubbles in unprecedented detail. The sonar systems were calibrated to allow for precise bubble size measurements, with *in situ* video recordings used for groundtruthing. The data were used to estimate gaseous methane bubble flux from the seabed. The presence of extensive gas ebullitions was subsequently validated in two additional surveys, in September 2017 and 2018, during weak storms with less pronounced water-level changes. As shallow gas accumulations are a common feature in coastal settings, our study suggests that storm-induced pressure fluctuations at the seafloor and subsequent gas ebullitions are a global phenomenon, but one that is likely to be missed in standard field campaigns.

Based on our sonar detections and calculations, we provide a first-order estimate of the methane flux for the Western Baltic Sea attributable to such ebullitions. Our report concludes with a comparison of our results to the methane fluxes estimated for ebullition hot spots^30,33,56^ and a discussion of the global implications of these findings.

## Results and Discussion

### A dedicated fine-tuned sonar survey strategy unveils extensive single gas ebullitions

**Figure 2:**
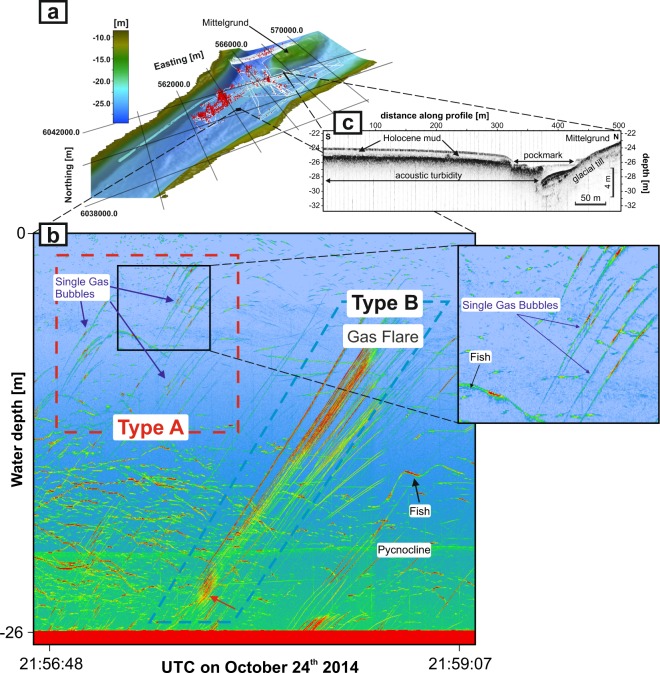
Spatial distribution and the diagnostic line pattern in the sonar representation of rising gas bubbles in the water column released from shallow gas in the Eckernförde Bay. (**a**) Overview of gas seepage locations and individual bubbles (red) in Eckernförde Bay, including the survey lines used for analysis (white). (**b**) Time-stacked multibeam echosounding (MBES) water-column imaging data showing the distinct pattern of inclined lines indicative of rising gas bubble tracks. The survey speed was <1 knot and the recording was made outside of a pockmark for 2.5 minutes. The fine-tuned sonar survey allowed the detection of Type A single rising gas bubbles; these were seen in most of the echograms and occurred in most parts of the bay. Type B refers to multiple gas bubbles (flares) in the water column; they occurred only in some locations. The quantitative analysis is based on split-beam echosounding (SBES) data on Type A, including single-echo detection for single targets (Methods). (**c**) Sediment echo-sounding profile crossing both the largest pockmark and the featureless Holocene mud. Note the extensive acoustic turbidity in the direction of the open bay, indicating shallow gas-bearing sediments.

Survey lines were sailed systematically, across shallow gas hosting areas in the bay, to avoid a bias in which ship-time would be preferentially spent at pockmarks (Figs. 1b and 2a). We found 2849 bubble seeps rising within 28 h from the seafloor across the 90 km² study site. Among the thousands of gas emissions detected, the vast majority were single gas bubbles occurring outside the pockmarks (Fig. 2b, Type A), but some had a flare-like appearance (Fig. 2b, Type B).

High-resolution sonar measurements of individual gas bubbles in the water column from shallow gas-bearing sediments have primarily been conducted in lakes^57–61^. Only a few hydroacoustic records have clearly shown individual bubbles in the ocean^62–65^.

At a very low sonar survey speed of approx. 1 knot per hour in Eckernförde Bay, the sonar’s high ping rate of 8 to 14 pings s^−1^ resulted in the large overlap of the acoustic beams, multiple detections and the tracking of single bubbles, and thus a much better detection reliability than achieved at the typically faster survey speeds during standard surveys. This novel survey strategy allowed for the identification of inclined track lines caused by rising individual gas bubbles (Fig. 2b, Type A) and enabled us to clearly discriminate them from those of fish and larger gas flares (Fig. 2b, Type B) as well as from the ubiquitous sonar interference (Supplementary Fig. S5). Data pattern Type A (Fig. 2) cannot usually be obtained at normal survey speeds and single gas bubbles are therefore overlooked; however, our unique dataset revealed the distribution of single gas bubbles throughout the bay and the presence of >2500 Type A individual gas bubbles within the 90 km² overall study area in 28 h (Fig. 2). More intense Type B sonar patterns (i.e. gas flares or plumes; Fig. 2b, Supplementary Fig. S4b and S7) were occasionally detected, but considering the sonar data shortages in the flux assessments of non-single targets, they were excluded from our methane flux calculations. While the actual flux is generally underestimated in methane flux calculations, our minimum flux, calculated based only on Type A single gas bubbles, can be considered robust. Two additional surveys in 2017 and 2018 validated the findings.

### Relation between seafloor morphology and gas ebullition

Except for a shallow morainic hill (Mittelgrund shoal, water depth <10 m, Figs. 1b and 2a) and three pockmark clusters, the ~21 to ~26-m-deep surveyed area was flat and featureless. Subbottom profiling during the survey (Fig. 1c) verified a shallow gas front depth of 50–75 cm below the seafloor (bsf), which is in line with earlier findings from this site^18,25,66,67^. The gas ebullition data did not provide evidence of a link between pockmarks and methane ebullition. Rather, ebullition, comprising both single bubbles and flares, was widespread across areas of the bay at sites where the seafloor consisted of organics-rich Holocene mud^44^ and at sites of shallow gas (Fig. 1). These findings suggest that areas prone to methane ebullition are orders of magnitude larger than previously considered and not restricted to methane emission hot spots such as pockmarks.

### Local methane flux estimate

The sediment-water methane flux of all methane ebullitions (F) was estimated, taking into account the number of Type A seep locations (2849) (#_loc_), the amount of methane per bubble (n_CH4_bubble_), and an ebullition frequency (f_e_):

$$F\,[Mol\,{s}^{-1}]={\#}_{loc}[-]\cdot {n}_{C{H}_{{4}_{bubble}}}\,[Mol]\cdot {f}_{e}[{s}^{-1}].$$

To assess a flux normalized for area and time, the sonar coverage area (A_ek60_) and time (T) of the sonar survey were calculated. The methane flux of all Type A seep locations (F) was normalized based on A_ek60_ [m^2^] and T [d] in order to estimate an areal methane flux from the sediment (F_area_ [μmol m^−2^ d^−1^]) of the entire surveyed area. The areal flux was then used to calculate the methane flux rates for the mean, minimum and maximum of both bubble size and ebullition frequency. Using this method, the sediment-water methane flux in the bay ranged from 981 to 3311 μmol m^−2^ d^−1^ (Table 1). The coverage-normalized methane flux was thus significant compared to the flux values reported in the literature for Type B hot spots at other locations (Table 2).

**Table 1:** Methane flux compilation for different mean bubble radii (a) and ebullition frequencies (f_e_). The extrapolation is based on areas of shallow gas given in Mogollon *et al*.^88^.

	This study: Eckernförde Bay								Extrapolation		
	Total Seeps [#]	Bubble radius [mm]	Bubble rate [# s^−1^]	Volume per seep [cm^3^ s^−1^]	Areal flux [mg m^−2^ d^−1^]	Areal flux [μMol m^−2^ d^−1^]	Total flux from all seeps [10^−4^ Tg y^−1^]	Covered Area [km^2^]	Belt Seas & The Sound [km^2^]	Extrapolated Flux [Tg y^−1^]	15 events same magnitude [Tg (15*24 h)^−1^]
**A. Best estimate**											
a_mean_, f_e_mean_	2849	3.75	0.25	0.0552	30.737	1916.3	0.1010	0.90	1602	0.270	0.011
a_min_, f_e_mean_	2849	3	0.25	0.0283	15.737	981.13	0.0517	0.90	1602	0.138	0.006
a_max_, f_e_mean_	2849	4.5	0.25	0.0954	53.113	3311.3	0.1746	0.90	1602	0.466	0.019
**B. Shift to max rate**											
a_mean_, f_e_max_	2849	3.75	0.5	0.1105	61.474	3832.5	0.2020	0.90	1602	0.539	0.022
a_min_, f_e_max_	2849	3	0.5	0.0565	31.475	1962.3	0.1034	0.90	1602	0.276	0.011
a_max_, f_e_max_	2849	4.5	0.5	0.1909	106.23	6622.6	0.3491	0.90	1602	0.932	0.038
**C. Shift to min rate**											
a_mean_, f_e_min_	2849	3.75	0.0833	0.0184	10.246	638.75	0.0337	0.90	1602	0.090	0.004
a_min_, f_e_min_	2849	3	0.0833	0.0094	5.2458	327.04	0.0172	0.90	1602	0.046	0.002
a_max_, f_e_min_	2849	4.5	0.0833	0.0318	17.704	1103.8	0.0582	0.90	1602	0.155	0.006

**Table 2:** Methane flux compilation as reported in the literature for different study sites for different transport mechanisms.

Reference	Location	Transport mechanism	Area [km^2^]	Seeps [n]	Flux^2^ [µMol m^−2^ d^−1^]
Artemov *et al*. (2007)^98^	Black Sea, Dnieper paleo-delta	gaseous, sediment	387	2200	5308^b^
Borges *et al*. (2016)^12^	Belgian coastal zone, near shore	diffusive, sea-air			130
Bussmann & Suess (1998)^99^	Baltic Sea, Eckernförde Bay	diffusive, sea-air			72
Hovland *et al*. (1993)^100^	USA, California, Coal Oil Point	gaseous, sediment	18	>900	68322
Leifer & Judd (2015)^37^	North Sea, UK22/4b	gaseous, sediment	0.04		125 × 10^6 b^
Martens & Klump (1980)^11^	USA, Cape Lookout Bight	diffusive, sediment	1.00		3912
Sahling *et al*. (2009)^64^	Black Sea, Sorokin trough	gaseous, sediment	0.02	1500^a^	115115^b^
Schneider von Deimling *et al*. (2011)^31^	North Sea, Tommeliten	gaseous, sediment	0.12	735	34247^b^
Shakhova *et al*. (2014)^30^	East Siberian Arctic Shelf	gaseous, sediment	18400	18506	4473
Skarke *et al*. (2014)^56^	Northern US Atlantic margin	gaseous, sediment	94000	~570	0.2^b^
**This study, best estimate**	**Baltic Sea, Eckernförde Bay**	**gaseous, sediment**	**0.90**	**2849**	**1916**
**This study, max estimate**	**Baltic Sea, Eckernförde Bay**	**gaseous, sediment**	**0.90**	**2849**	**3311**
**This study, min estimate**	**Baltic Sea, Eckernförde Bay**	**gaseous, sediment**	**0.90**	**2849**	**981**
Washburn *et al*. (2001, 2005)^40^	USA, Coal Oil Point	gaseous, sediment	3.00		2.56 × 10^6 b^
Bange *et al*. (1994)^14^	Global continental shelves	diffusive, sea-air			30
Rhee et al. (2009)^101^	Open oceanic waters	diffusive, sea-air			0.4

^a ^bubbles per minute.

^b ^value derived from information provided in the original work.

Geochemical sampling during the survey revealed exceptionally high dissolved methane concentrations of up to 800 nmol L^−1^ in a bottom water layer of up to 3 m above the seafloor, which is >250-fold oversaturated with respect to the atmospheric equilibrium (≤3 nmol L^−1^ at present temperature and salinity^68^). Methane oxidation (MOx) rates at Eckernförde Bay are typically <10 nmol L^−1^ d^−1^^10^, which translates to a methane turnover time of more than 10 days. During spring and summer, Baltic Sea basins like Eckernförde Bay are characterized by a thermohaline stratification that impedes vertical mixing and a rapid transportation of methane to the sea surface leading to the formation of methane reservoirs below these density barriers^69^. However, wind-stirring and thermal convection, especially during autumn and winter time, promotes the transportation of methane to the sea surface within time scales of hours^30^ that may outcompete an efficient pelagic microbial methane turnover and result in enhanced atmospheric methane fluxes^69^. Based on the bubble dissolution model of McGinnis *et al*.^9^, we assume that at least 45% of the initial methane content of the bubbles reaches the sea surface, whereas the rest remains in the water column. Water samples taken on a monthly basis at nearby Boknis Eck since 2006 show a yearly averaged surface methane saturation of approx. 520%^70^, also providing evidence that a substantial fraction of dissolved methane is liberated to the atmosphere^69,70^. Still, a contribution of pelagic methane sources like phytoplankton^71,72^ or zooplankton^73,74^ to the observed supersaturation within the oxic waters of the Baltic Sea is likely. Even if not studied in detail in the present field campaign, it might be speculated that the dissolved methane plume is transported out of the bay by currents and partially mixed into the surface waters and liberated to the atmosphere.

### Impact of seasonal dynamics on gas ebullition

Methane ebullitions in Eckernförde Bay most likely occur mainly during the fall, as during spring and summer the storm activity is low and both the water and sediment temperature in shallow waters begin to rise. The warmer temperatures accelerate microbial methanogenic activity while lowering methane solubility. They also intensify stratification of the water column and support the development of phytoplankton blooms in early spring and autumn^70,75,76^. The decline of these blooms is accompanied by strongly increased rates of organic matter transport to the seafloor, leading to hypoxic conditions in the bottom water and enhanced methanogenesis at shallow sediment depths^48,69,70,77^. By late fall, seabed temperatures peak and potentially drive free methane gas close to the seafloor at 0.5 mbsf. This minimum depth of the shallow gas front^66^ likely correlates with a maximum in the amount of sub-seabed methane.

Seiches due to lasting westerly winds may interrupt methane accumulation by inducing significant fluctuations in the hydrostatic pressure^78^. The ensuing ebullition is presumably pressure-induced as reproduced in laboratory studies^79,80^. The Type B methane flares seen in a scuba-diver video recorded in Eckernförde Bay in 2013 support this mechanism and provide visual evidence of particularly intense ebullition during strong storm events^81^ (Supplementary Figs. S7 and S8). Specifically, the recording shows high frequencies of ebullition and the release of larger groups of bubbles (Type B) often surrounded by individual gas bubbles (Type A) released in parallel (Supplementary Fig. S7).

Although the data used in our methane flux estimates were acquired during a weak storm in the fall of 2014 (Supplementary Fig. S6), intense methane ebullition was also observed 3 and 4 years later, during an even weaker wind event in which the maximum wind speed never exceeded 8.5 m/s. Since strong winds cause more intense changes in hydrostatic pressure, stronger and more frequent gas ebullitions can be expected during more powerful storms, as evidenced by the video footage from the bay taken during a storm in autumn 2013^81^ (Supplementary Figs. S7 and S8). We conclude that the benthic filter and oxidation of methane in the sediment is temporarily bypassed by extensive methane ebullition of substantial magnitude.

### The problems of discrete sampling, event detection, and a focus on methane emission hot spots

Sonar bubble detection has been conducted successfully in the ocean since decades^82^, including its recent application to identify ebullition hot spots from biogenic or thermogenic sources down to a water depth of 2800 m^83^. Most marine methane ebullitions are characterized by a clustered occurrence of gas bubbles in the water column at locally constrained seep sites (Type B, flares). However, due to nautical constraints, many ebullition events occurring during stormy weather, such as those described herein, would be missed^31^.

The broad release of methane bubbles from the sediments of Eckernförde Bay demonstrated by our dataset is especially noteworthy given that major research campaigns investigating sedimentary methane emissions in this region did not obtain conclusive evidence of methane ebullition^84–87^.

The failure to detect extensive ebullition from shallow areas during decades of research has several potential explanations: (1) marine survey logistics hardly allow for sailing-on-demand to study episodic events; (2) the failure to detect Type A single gas bubbles in shallow waters at survey speeds higher than the very slow speed (approx. 1 knot) of this study; (3) the difficulty in acquiring high-quality sonar data during storms; (4) enhanced surface water mixing and air-sea exchange during storms hinders the accumulation, and therefore the detection, of dissolved methane in the water column; (5) the comparative ease of detecting strong and permanent Type B methane seeps during typical surveys, with their higher speeds; and (6) the lack of an association between Type A seepage and acoustic seepage indicators, such as pockmarks, faults, and/or chemosynthetic communities, all of which generally guide seepage detection during regular seabed mapping. Low to near-stationary survey speeds and high ping rates are essential to unambiguously image minor single bubble methane ebullitions in the water column. This requires a dedicated strategy with lowest possible acoustic noise and time-intensive drift surveys using high-resolution sonars at high ping rates.

### Extrapolation from a local flux to a flux for the Western Baltic Sea and global implications

Our estimates of methane flux are conservative because they were based on Type A single bubble tracking, which is a much more reliable approach than acoustic estimation of methane flux from seeps with unknown bubble sizes and uncertain sampling geometries (Fig. S2/3).

Changes in the water level and atmospheric pressure during storms are of similar magnitudes throughout the Baltic Sea^78^ and induce hydrostatic pressure changes. Accordingly, similar ebullition rates in all Holocene mud areas in shallow Eckernförde Bay (~80 km²) would yield a net methane flux of 1916 μMol m^−2^ d^−1^ (Table 1, best estimate). The occurrence of massive Type B ebullitions outside the surveyed area is supported by the above-mentioned video footage from 2013 (Supplementary Fig. S7), taken during a stronger storm event (Supplementary Fig. S8) when the bubble release frequency was significantly higher^81^ than that concluded from the sonar data of our study (f = 10 Hz vs. f = 0.02–0.1 Hz, respectively).

Gassy Holocene sediments are not restricted to Eckernförde Bay. For example, Mogollón *et al*. estimated that shallow gas-hosting sediments cover an area of 5113 km² in the Belt Sea and 509 km² in the Danish Sound at water depths <35 m^88^ and very similar geological/oceanographic settings (Fig. 1a). Based on meteorological data from a lighthouse close to the survey area in 2014, we found 15 events during which wind speeds exceeded 8 m/s (5 Bft) for 24 hours. Water level data from the same station documented 15 events per year during which the water level dropped significantly with a rate of 5 cm/h or faster in the years 2005 to 2018, further pointing towards the event-based nature of ebullition. Assuming 15 Type A ebullition events per year under similar meteorological and oceanographical conditions and that the events were of the same magnitude as in Eckernförde Bay, then the annual methane flux would be 110×10^−4^ Tg (best estimate) from the Belt Sea and the Danish Sound. Thus, the magnitude of yearly methane emissions in this small area of the Baltic Sea would be similar or stronger than Type B methane emissions from prominent seepage hot spots such as Tommeliten (North Sea, 15×10^−4^ Tg per year). Estimates of methane flux emissions in the open ocean are mainly derived from such thermogenic seepage sites. However, these are characterized by locally constrained Type B ebullitions and do not consider the potentially high amounts of Type A individual methane bubbles emitted from the surrounding gas-bearing sediments.

The kinetics of microbial methanogenesis in shallow sediments depend on several parameters, including oxygen supply, sulfate concentration gradients, temperature, and the availability of methane precursors. As these parameters vary substantially on a global scale, their impact on gas ebullition needs to be addressed in further studies. As long as site-specific prerequisites and triggers of marine seepage are not yet fully understood, we have to assume that vast areas of the continental shelf have at least the potential for unnoted marine seepage events as presented in this study. Literature proves that coastal shallow gas is a widespread phenomenon around the globe^19^ even though sedimentation regimes are fundamentally different for many areas.

Marine areas with water depths <35 m worldwide cover 7.87×10^6^ km² ^89^. In mapping the extent of hydrocarbon-bearing sedimentary basins, St. John determined that they occupy a significant proportion (10–30%) of the continental shelves^90^. It was later suggested that all such basins are potential sites of methane ebullition^91^. Fleischer *et al*. compiled studies on methane-charged seafloor sediments and showed that they occur in many parts of the world even though this compilation is far from complete^19^. Kortekaas *et al*. estimated that 25% of deltaic sediments, and especially those in temperate and hot, humid environments hold shallow gas^92^. Assuming that 10% (in analogy to the Belt Seas and the Danish Sound^88^) of the global <35 m^12^ shelf areas (≥45% of initial methane content reach the atmosphere for bubble sizes found in this study)^9,12^ contain shallow gas would lead to a 500-fold area of potential ebullition sites compared to the Belt Seas and the Danish Sound. Storms occur in the Baltic Sea ~15 times a year, and similar activity can be expected at higher latitudes on a global scale. Thus, the potential methane release from these worldwide shallow shelf areas for comparable ebullition events as described in this study would be in the same order of magnitude as estimated for the marine Arctic permafrost^30^ or the northern US Atlantic margin^57^. Even if a portion of the methane released from the seabed is dissolved and consumed by methanotrophic bacteria in the overlaying water column, the largest amount of methane in these shallow coastal regions will presumably still be mixed into the atmosphere^30^. Alternative drivers of changes in hydrostatic pressure, especially in shallow coastal areas, are tidal amplitudes, with maxima occurring every 2 weeks. The tidal triggers of methane ebullition in coastal areas have yet to be systematically studied and how long it takes to refill shallow gas reservoirs after extensive gas ebullition is unclear.

## Conclusion and Outlook

Storm-associated and therefore event-driven methane ebullitions can be easily overlooked by the ‘standard’ surveying strategies of marine acoustic research. However, our fine-tuned sonar survey strategy allows for unmatched detection of weak but widespread methane ebullition in shallow waters. It enables a quantitative and robust assessment of gaseous methane flux, based on acoustic single target detection and diagnostic line pattern recognition and the counting of individual gas bubbles along the survey track. While the extrapolated methane flux for the Western Baltic Sea calculated herein is not definitive, it does provide a conservative estimate that draws attention to the role played by event-driven methane emissions from coastal shallow gas reservoirs. Consequently, future research focusing on gas bubble methane flux from the seabed to the atmosphere should target extensive shallow water gas-bearing regions instead of deep water hot spots. Hot, humid and temperate delta systems, such as in the tropics, may be another significant source of methane but their impact has yet to be quantified^29^. Verification by continuous monitoring or an event-driven research campaign, possibly supported by the use of unmanned aerial or underwater vehicles, would lead to improved global estimates. In addition, closer cooperation with authorities, companies and research institutions with active interests in coastal zones should be expanded, with the long-term aim of creating a global database for seabed shallow gas that could be used to obtain more accurate estimates of methane flux.

Numerical models project that during the 21^st^ century deoxygenation will continue^93^, caused by rising seawater temperatures, a decrease in oxygen solubility, an increase in stratification, and the weakening of ocean turnover^94,95^. These developments will enhance the rate of microbial oxygen consumption in the water and in surface sediments, thus favouring biogenic methane production and the accumulation of methane in the seabed^29^. Given the dependency of ebullition on temperature^32^, a steep increase in methane release from the seafloor to the atmosphere can be anticipated.

## Methods

### High-resolution seafloor mapping

A Kongsberg EM2040c multibeam echosounder (MBES) was installed in the moonpool of the R/V ALKOR, which minimized motion of the device while also circumventing bubble wash-down and acoustic damping during rough sea states at 4.6 m sonar draft depth. The system was operated at a base frequency of 300 kHz with a pulse length of 50 μs at a 130° opening angle. Keel sound velocity, GPS positioning, and vessel motion were measured and provided to the MBES in real-time using a Codaoctopus F180R inertial navigation and motion reference unit (MRU), whose offsets to the transducer had been measured. The MRU was fully calibrated and its positioning was aided by the reception of EGNOS correction data with a positioning accuracy of close to 0.5 m. The data (Fig. S1) were processed using MB-System software; yaw, pitch, and roll offsets were calibrated using dedicated patch-test lines. Time latency was not calibrated because pulse-per-second (PPS) timing was provided. Bathymetry was re-calculated using sound velocity profiles (SVP) from five different CTD casts to minimize residuals and to apply ideal sound refraction. Due to the strong effect of short-term water-level fluctuations in shallow waters during the survey, tidal reduction based on the water levels of a near-by gauge station was implemented as a correction.

### Sub-bottom profiling

An Innomar SES-2000 standard parametric sediment profiler was used along the N-S and W-E survey lines to gather information on sub-bottom structures near the largest pockmark cluster close to Mittelgrund shoal (Fig. S1). The system produced parametric secondary frequencies between 5 and 10 kHz and motion compensation was provided by a Kongsberg Seatex MRU-M-MB 1.

### Gas bubble detection and inversion of the target strength to the bubble radius

We sailed 93 nautical miles across the bay to detect rising gas bubbles in the water column on a grid spanning 2 × 4 nautical miles (Fig. 1). Survey lines were designed to cover both pockmarks and featureless Holocene organics-rich mud hosting shallow gas. The MBES was used to map the pockmarks and a KONGSBERG Simrad EK60 split-beam echo sounder was used to monitor the water column during the survey. The split-beam system was operated at four frequencies (38, 70, 120, and 200 kHz). The 70-kHz data with a −3 dB opening angle of α = 6.87° and a pulse length of 256 μs were most sensitive to gas bubbles in the water column and were therefore used in the analyses. The system was calibrated for single-target detection by lowering a standard copper sphere of known target strength (TS) below the transducer and moving it in a raster using a winch driven setup (Fig. S2). The system uses a split-beam configuration to obtain precise information on the sphere’s position based on phase shifts.

The analysis of the water column data focused on the inclined track patterns seen in the amplitude and single-echo detection (SED) echograms. These patterns are diagnostic of Type A individual gas bubbles, due to the indicated buoyancy (Fig. S3). It was therefore possible to clearly discriminate the bubbles from the swim bladders of fish^58–60^. Sonar5-Pro, a commercial fisheries software, was used to visualize the amplitude and SED echograms. To eliminate passive noise in the water column, the display thresholds in both sets of echograms were set to above −85 dB for amplitude echograms and −90 dB for SED echograms, following the method of^58^.

Candidate gas bubbles in the SED echograms were manually selected to access SED target information. The software allows for the evaluation of SED tracking point positions in the XY (Fig. S3a) and XZ (Fig. S3b) planes of the selected candidate bubbles. This feature was used to analyse the candidate bubbles’ movements inside the −3 dB lobe of the acoustic beam. Subsequent numerical filters on SED information confirming the smoothness and upwards movement of the candidate bubble validated the manual selection. The candidate bubble was accepted and classified as a gas bubble if monotonic upwards movement (>1 m), rise velocity (−0.1 > V_z_ > −0.5 m/s), TS (−50 dB> TS > −65 dB) and sufficient smoothness (>0.85) in both the lateral XY and the vertical XZ planes was confirmed. In these cases, the SED information was stored for later assessment^59^. Candidate bubbles that failed to meet the above criteria were dismissed. This routine ensured the unambiguous discrimination of gas bubbles and upward swimming fish.

Gas bubble identification was implemented in two steps: (i) visual identification and manual selection of candidate rising gas bubbles by switching between amplitude- and SED-echogram-based visualization as well as XY and XZ plane views of the SED tracking points to verify monotonic upwards movement (Fig. S3) and (ii) subsequent numeric filtering of the SED information of the candidate gas bubble with respect to the above-described criteria.

Our method was used for Type A seepage because an acoustic flux assessment of Type B seepage is possibly unreliable. When larger amounts of bubbles leave the seafloor at the same time, neither phase separation techniques nor single-target echo-pulse shape detection can be applied. Therefore, the relation between beam geometry and gas volume remains unclear and quantitative estimates accordingly compromised. The non-linear acoustic behavior and resonance phenomena that occur when the bubble size spectra are unknown lead to ambiguous results and errors that cannot be quantified. Acoustic sensing of Type A seepage in shallow water does not suffer from these drawbacks. In our study, if a single pulse suggested the presence of multiple bubbles, the candidates were always classified conservatively as one gas bubble, such that the minimum number of bubbles was estimated. In addition, where possible, candidate gas bubbles were selected in low oxygen zones, to minimize the risk of erroneously including fish swim bladders.

Due to accessibility limitations, we chose a subset of targets classified as gas bubbles based on >30 SED tracking points (Fig. S3) per gas bubble for the inversion of the TS to the bubble radius. Knowledge of the resonance frequency (*f*_r_) of the gas bubbles, and therefore of the mean bubble radius (*a*_est_) and mean water depth (*z*), is required for the inversion (Eq. 1). In this study, *z* = 25 m and *a*_est_ = 3 mm.

1 $${f}_{r}=\frac{3.25\,\sqrt{1+0.1z}}{{a}_{est}}$$

Resonance frequency approximation (after Medwin & Clay^96^).

To invert from the measured TS to backscattering cross-section (${\sigma }_{{\rm{bs}}})$, we used Eq. 2 to define the TS of a single bubble:

2 $$TS=10\cdot lo{g}_{10}{\sigma }_{{\rm{bs}}}$$

Target strength (TS) of a single bubble (after Clay & Medwin^97^).

and then rearranged the terms to obtain the mean backscattering cross-section of a single bubble, as shown in Eq. 3:

3 $${\sigma }_{{\rm{bs}}}={10}^{\frac{TS}{10}}.$$

Backscattering cross-section of a single bubble.

Assuming that the shape of a small rising gas bubble is close to a sphere, its mean backscattering cross-section for a spherical equivalent radius ${a}_{{\rm{bubble}}}$ for the known sonar frequency ($F$) can be described by Eq. 4:

4 $${\sigma }_{{\rm{bs}}}=\frac{{a}_{bubble}^{2}}{{[{(\frac{{f}_{{\rm{r}}}}{f})}^{2}-1]}^{2}+{d}^{2}}$$

with $$d=0.0025\cdot \sqrt[3]{f}$$

The backscattering cross-section of a spherical single bubble (after Clay & Medwin^97^).

the terms of which can be rearranged to estimate the gas bubble radius:

5 $${a}_{{\rm{bubble}}}=\sqrt{{\sigma }_{bs}}\cdot \sqrt{{[{(\frac{{f}_{r}}{f})}^{2}-1]}^{2}+{d}^{2}}.$$

Bubble radius estimated from the backscattering cross-section.

The equations are valid only for $ka\ll 1$ (where $k$ is the corresponding wave number) and an estimated damping factor $d$. For the sonar frequency of our study $ka$ was 0.14–0.21. The resulting uncertainty has to be taken into account.

### Bubble radius to gas flux estimation

The estimated gas bubble radius (${a}_{{\rm{bubble}}}$) served as input for the calculation of the methane mass of a single gas bubble transported through the water column (m_CH4_bubble_), assuming standard pressure (P) and temperature (T) conditions and applying the ideal gas law for the individual gas constant of methane (R_S_), as shown in Eq. 6:

6 $${m}_{C{H}_{{4}_{bubble}}}[g]=\,\frac{P[Pa]\cdot V[{m}^{3}]}{{R}_{s}[\frac{J}{kg\cdot K}]\cdot T[K]}$$

Total mass of methane per gas bubble.

where bubble radius ($a$), volume of the bubble ($V$), universal gas constant ($R$), specific gas constant of methane (${R}_{s}$), temperature ($T$) are defined as:

$$a[m]=0.003\ldots 0.0045$$

$$V[{m}^{3}]=\frac{4}{3}\pi {r}^{3}$$

$$R=8.31448\frac{J}{Mol\,K}$$

$${R}_{s}=\frac{R}{{M}_{C{H}_{4}}}=\frac{8.3144598\,\frac{J}{Mol\,K}}{16.04\,\frac{g}{Mol}}=518.4\frac{J}{kg\cdot K}$$

$$T=287K=14\,^\circ {\rm{C}}\,({Water}\,{temperature}\,{close}\,{to}\,{sediment})$$

7 $${n}_{C{H}_{{4}_{bubble}}}=\frac{{m}_{C{H}_{{4}_{bubble}}}}{{M}_{C{H}_{{4}_{bubble}}}}$$

Mole amount of methane per gas bubble.

Assuming a mean water depth of 20 m, the effective pressure (P) acting on the gas bubble is the sum of the atmospheric pressure (P_atm_) and the hydrostatic pressure (P_hyd_) due to the overlying water column (Eq. 8):

8 $$P={P}_{atm}+{P}_{hyd}$$

Total pressure at the seafloor.where

$${P}_{atm}[hPa]=1.05{e}^{5}$$

and

$${P}_{hyd}[hPa]={\rho }_{water}[\frac{kg}{{m}^{3}}]\cdot g[\frac{m}{{s}^{2}}]\cdot {h}_{water}[m]=1.03\frac{kg}{{m}^{3}}\cdot 9.81\frac{m}{{s}^{2}}\cdot 20\,m=2.02{e}^{5}.$$

The methane flux of all gas seep locations was estimated, taking into account the number of seep locations (2849) (#_loc_), the amount of methane per gas bubble (n_CH4_bubble_), and a gas bubble emission frequency (f_e_), as shown in Eq. 9:

9 $$F\,[Mol\,{s}^{-1}]={\#}_{loc}[\,-\,]\cdot {n}_{C{H}_{{4}_{bubble}}}\,[Mol]\cdot {f}_{e}[{s}^{-1}].$$

Total methane flux of all seep locations for single methane bubbles.

To determine a normalized flux with respect to area and time, the coverage and time of our acoustic mapping were calculated. Seafloor coverage is dependent on the opening angle of the system (α), the water depth (z), and the survey length. The acoustic footprint of the system was calculated for each ping inside the survey area. To compensate for overlap, the coverage was adjusted by calculating the overlap of all successive pings based on the footprint size and distance between the pings, resulting in an effectively surveyed seafloor coverage (${A}_{ek60}$).

The methane flux of all seep locations ($F$) was normalized to the effectively surveyed seafloor coverage (${A}_{ek60}$) in order to estimate an areal methane flux (${F}_{area}$) for the whole survey area (Eq. 10):

10 $${F}_{area}[Mol\,{s}^{-1}{m}^{-2}]=\frac{F\,[Mol\,{s}^{-1}]}{{A}_{ek60}\,[{m}^{2}]}.$$

Areal methane flux.

${F}_{area}$ was then used to calculate the flux rates for mean, minimum and maximum gas bubble radii and emission frequencies. The results are reported in Table 1.

### Geochemical sampling

We collected water column samples with 5 L Niskin bottles mounted on a CTD/Rosette sampler and subsampled the Niskin bottles immediately upon recovery for on-board measurement of CH_4_ concentrations using a headspace method. For this, 120 ml aliquots were collected bubble-free into 120 mL glass vials. The samples were fixed with 5 mL of aqueous NaOH solution (30%, w/v) and 5 mL of high-purity nitrogen as a headspace were added. The samples were shaken, and allowed to equilibrate for several hours before subsampling of 200 μL of headspace from the sample for CH_4_ quantification with a gas chromatograph (Hewlett Packard 5890 GC) equipped with packed stainless steel column (1.83 m length, 2 mm i.d., 80/100 mesh HayeSep Q) and a flame ionization detector. The gas chromatograph was operated isothermally at 60 °C with N_2_ as carrier gas (30 mL min^−1^). The gas chromatograph system was calibrated with methane standards (10, 100 and 1000 ppm) and the analytical error (determined from triplicate subsamples) was <±5%.

## Supplementary information

Supplementary Information.

## Data Availability

The datasets generated and analysed during the current study are available from the corresponding author on reasonable request.
