# Supplementary material for: Discovery and quantification of a widespread methane ebullition event in a coastal inlet (Baltic Sea) using a novel sonar strategy
Source: Sci Rep. 2020 Mar 10;10:4393. doi: 10.1038/s41598-020-60283-0 (PMC7064498; doi:10.1038/s41598-020-60283-0)
Supplement: Supplementary file 1 — Supplementary Information. [file 41598_2020_60283_MOESM1_ESM.pdf]

Supplementary Information

Discovery and quantification of a widespread methane ebullition event in a coastal inlet (Baltic Sea) using a novel sonar strategy

Authors: A. Lohrberg[1], O. Schmale[2], I. Ostrovsky[3], H. Niemann[4], P. Held[1], J. Schneider von Deimling[1]*

*corresponding author J. Schneider von Deimling, Otto-Hahn-Platz 1, 24118 Kiel, Germany,
Tel. +49 431 880-5792, jens.schneider@ifg.uni-kiel.de

[1] **Christian-Albrechts-Universität zu Kiel**, Institute for Geosciences, Marine Geophysics & Hydroacoustics, Otto-Hahn-Platz 1, 24118 Kiel, Germany

[2] **Leibniz Institute for Baltic Sea Research Warnemünde**, Trace Gas Biogeochemistry, Seestraße 15, 18119 Rostock, Germany

[3] **Israel Oceanographic and Limnological Research**, Yigal Alon Kinneret Limnological Laboratory, Migdal, Israel

[4] **Royal Netherlands Institute for Sea Research**, Microbiology & Biogeochemistry, Landsdiep 4, 1797 SZ 't Horntje (Texel), the Netherlands

Author order:

1. Arne Lohrberg[1], arne.lohrberg@ifg.uni-kiel.de
2. Oliver Schmale[2], oliver.schmale@io-warnemuende.de
3. Ilia Ostrovsky[3], ostrovsky@ocean.org.il
4. Helge Niemann[4], helge.niemann@nioz.nl
5. Philipp Held[1], philipp.held@ifg.uni-kiel.de
6. Jens Schneider von Deimling[1], jens.schneider@ifg.uni-kiel.de

Supplementary Figures

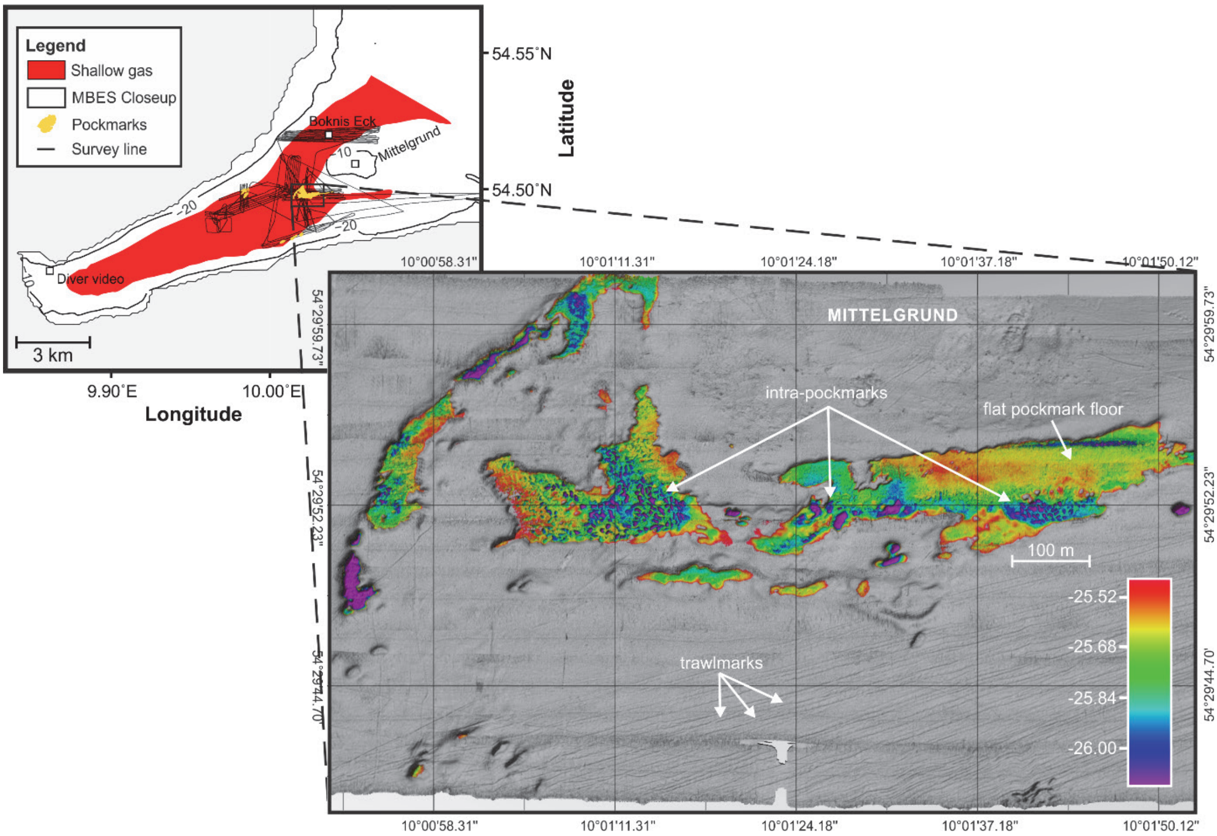

Figure S1 | High-resolution fully calibrated multibeam bathymetry of the pockmark southwest of the moraine outcropping shoal ‘Mittelgrund’. Data were recorded with a Kongsberg EM2040c at 300kHz during expedition AL447. The bathymetric data are presented in UTM32N projection and depth values outside 25.46 and 26.14 m are color coded in grey. This allows us to highlight what we term intra-pockmarks: round shaped depressions of a few meters diameter, possibly indicating active groundwater discharge. At the bottom of the image, lines appear striking southwest to northeast. They are 4 cm deep and are interpreted as fishery bottom trawl marks.

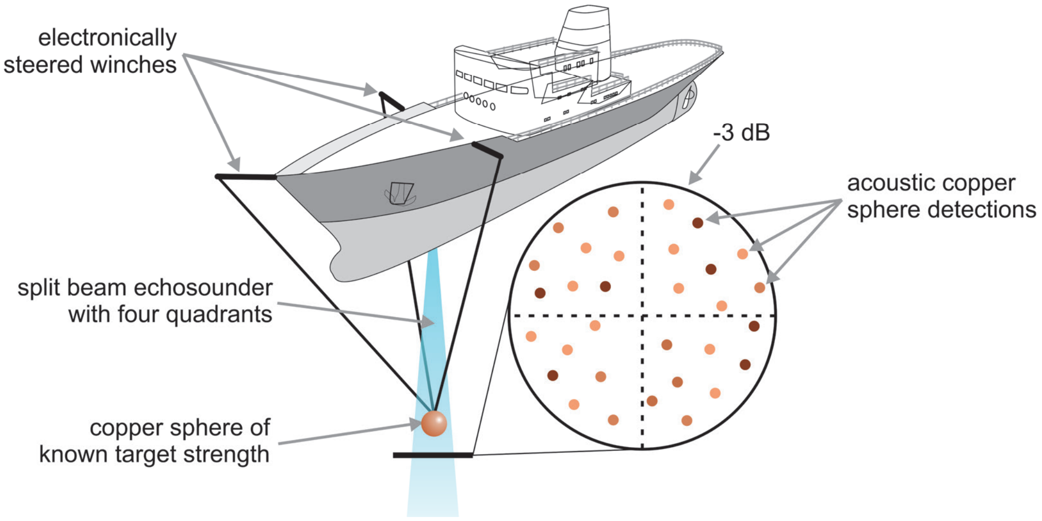

Figure S2 | Schematic drawing of the KONGSBERG Simrad EK60 split-beam system calibration performed during our expedition on R/V ALKOR with copper spheres of known target strength.

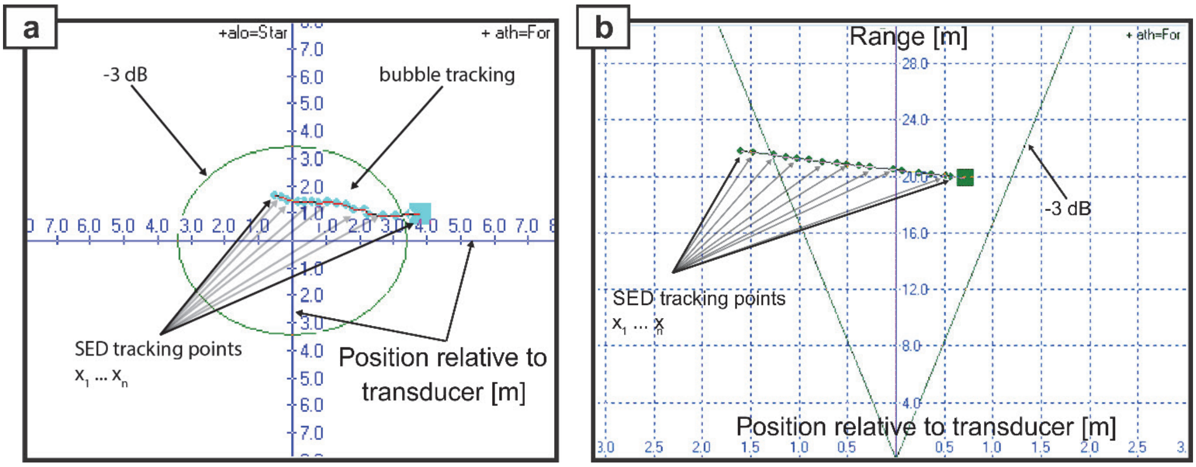

Figure S3 | Tracking of a single gas bubble based on single echo detection. (a) Lateral XY plane (deviation from center of beam, shown in meters) and (b) vertical XZ plane (range from the transducer, shown in meters). The big square indicates the last tracking point. All previous points contain detailed SED target information.

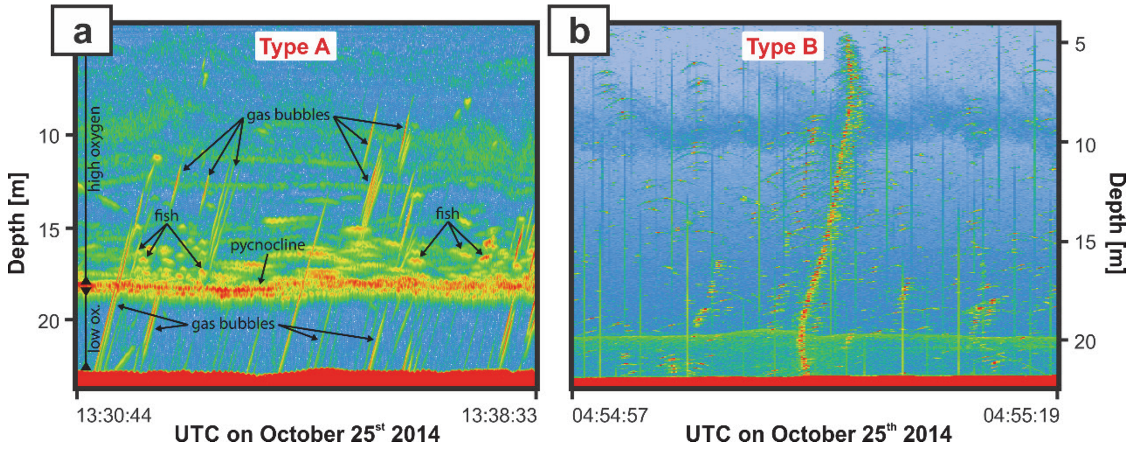

Figure S4 | Comparison of Type A and Type B ebullition. (a) Example of an amplitude echogram of a fine-tuned survey Type A rising gas bubbles emerging from the seafloor and penetrating the pycnocline. The swim bladders of fish can clearly be distinguished from gas bubbles due to the non-monotonic movement of the fish. (b) Example of flare-like Type B ebullition together with acoustic interferences (vertical lines) as seen in the MBES-water-column-imaging data.

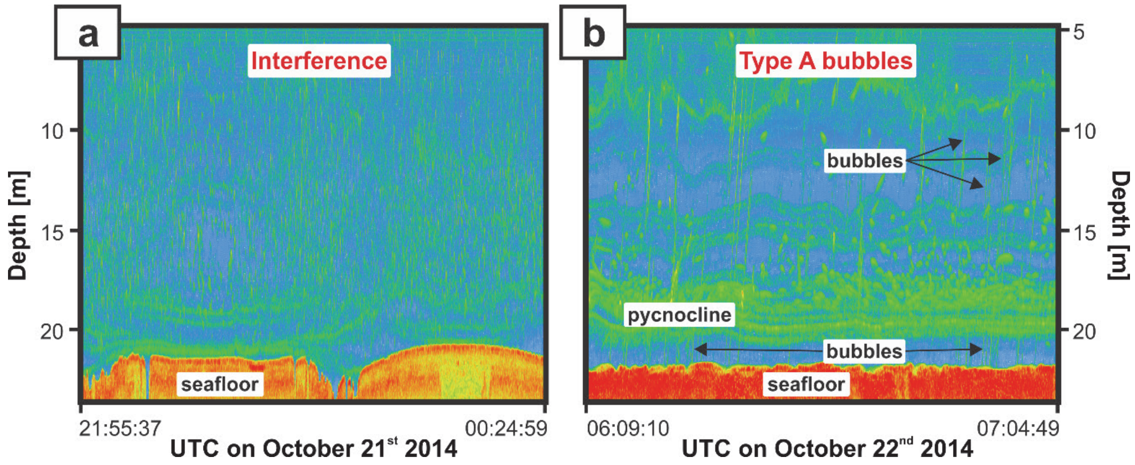

Figure S5 | Example of the difficulty in identifying minor gas bubble ebullition. (a) The echogram is overprinted by interfering acoustic signals appearing as rising features. (b) Gas bubbles appear nearly vertical and could be easily mistaken for interfering signals.

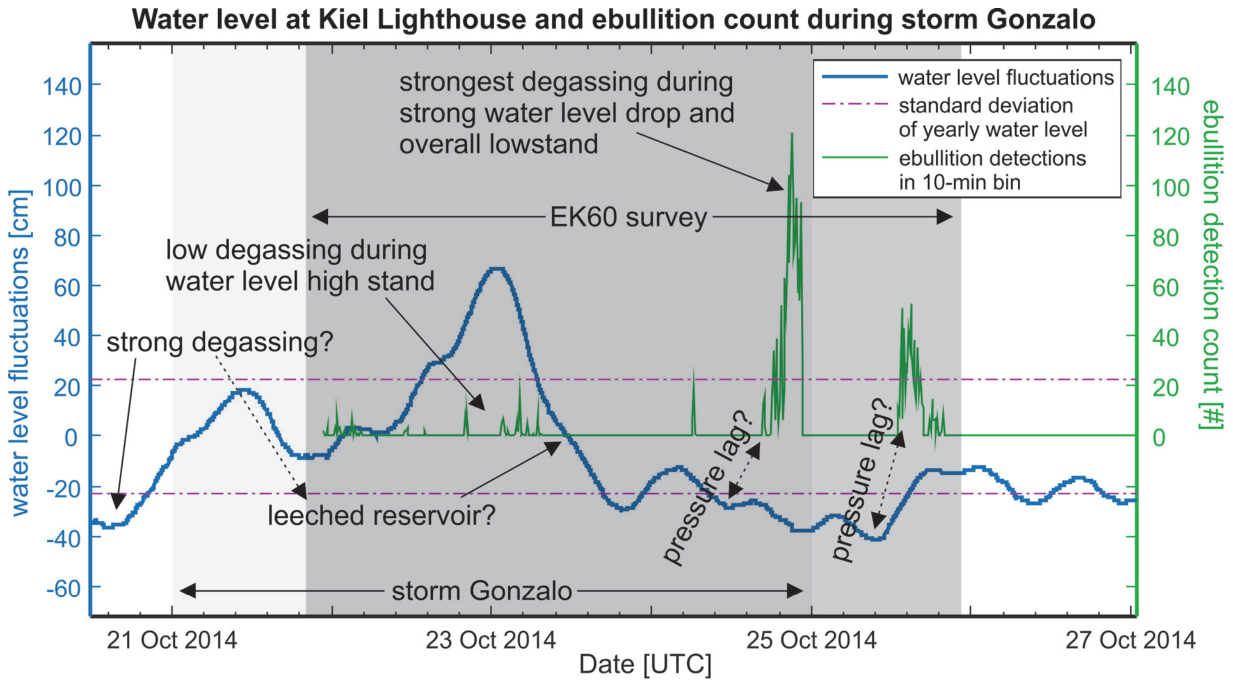

Figure S6 | Water level gauge time series nearby Kiel Lighthouse during the storm "Gonzalo" in 2014, enclosing our hydroacoustic assessments. Dedicated slow survey speeds for detection of minor gas seeps were only conducted during the "EK60 survey" time window for this storm. Although the ebullition time series is short, the water level indicates increased degassing during water level low stands and strong water level drop. Water level data kindly provided by the Wasserstraßen- und Schifffahrtsverwaltung des Bundes (WSV) and the Bundesanstalt für Gewässerkunde (BfG).

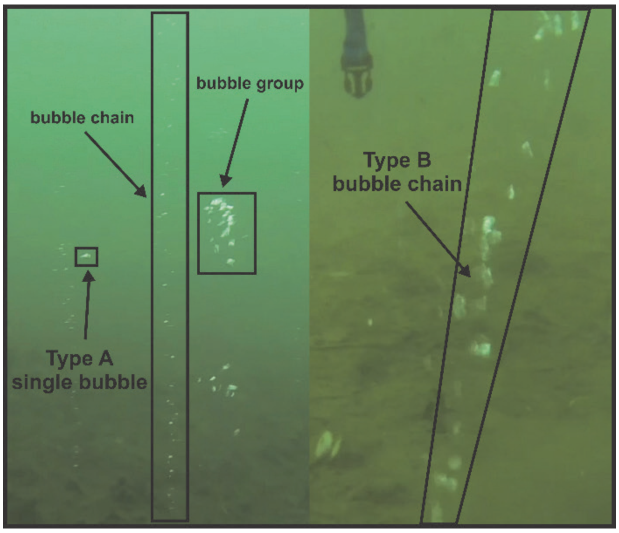

Figure S7 | Optical evidence of Type B multiple gas bubble ebullition with a major flux, as well as Type A single bubbles [95]. The video footage was recorded on October 28, 2013 by Sönke Staack

during an intense low-pressure system (Fig. S8). The complete video is available online at https://youtu.be/mcwBMT6vqlA.

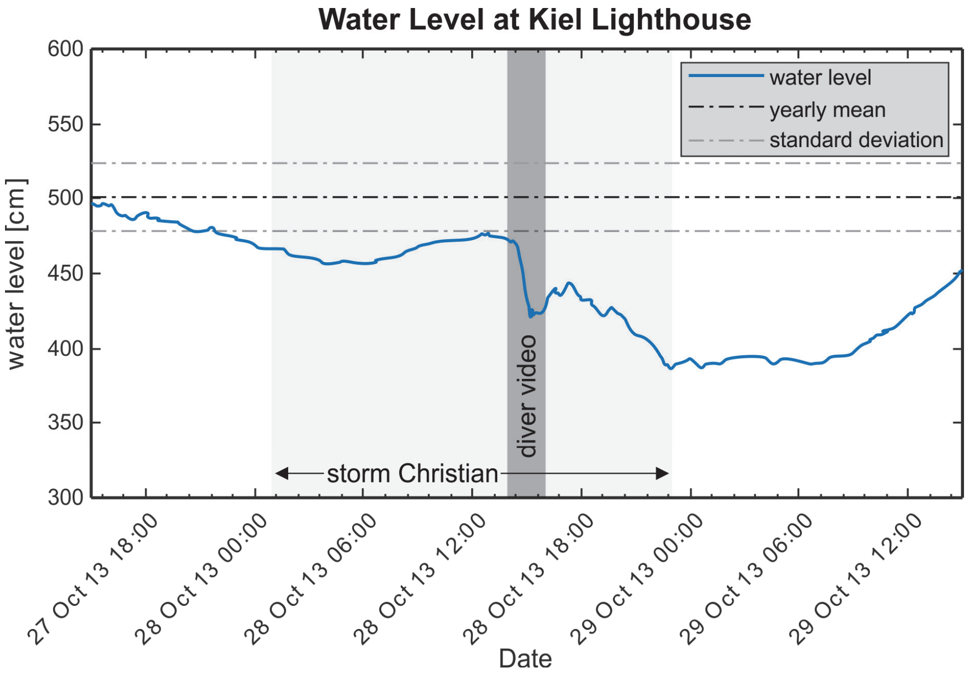

Figure S8 | Water level gauge time series nearby Kiel Lighthouse during the storm "Christian" in 2013. The recording of the diver video is indicated and corresponds to a significant water level drop induced by the storm.
